# Naloxone inhibits immune cell function by suppressing superoxide production through a direct interaction with gp91^*phox *^subunit of NADPH oxidase

**DOI:** 10.1186/1742-2094-9-32

**Published:** 2012-02-16

**Authors:** Qingshan Wang, Hui Zhou, Huiming Gao, Shih-Heng Chen, Chun-Hsien Chu, Belinda Wilson, Jau-Shyong Hong

**Affiliations:** 1 Neuropharmacology Section (Q.S.W., H.Z., H.M.G., S.H.C., C.H.C., B.W., J.S.H.), Laboratory of Toxicology and Pharmacology, National Institute of Environmental Health Sciences, Research Triangle Park, NC, USA

**Keywords:** Neuroinflammation, Microglia, NADPH oxidase, Opioid receptor, Binding

## Abstract

**Background:**

Both (-) and (+)-naloxone attenuate inflammation-mediated neurodegeneration by inhibition of microglial activation through superoxide reduction in an opioid receptor-independent manner. Multiple lines of evidence have documented a pivotal role of overactivated NADPH oxidase (NOX2) in inflammation-mediated neurodegeneration. We hypothesized that NOX2 might be a novel action site of naloxone to mediate its anti-inflammatory actions.

**Methods:**

Inhibition of NOX-2-derived superoxide by (-) and (+)-naloxone was measured in lipopolysaccharide (LPS)-treated midbrain neuron-glia cultures and phorbol myristate acetate (PMA)-stimulated neutrophil membranes by measuring the superoxide dismutase (SOD)-inhibitable reduction of tetrazolium salt (WST-1) or ferricytochrome c. Further, various ligand (^3^H-naloxone) binding assays were performed in wild type and gp91*^phox-/- ^*neutrophils and transfected COS-7 and HEK293 cells. The translocation of cytosolic subunit p47*^phox ^*to plasma membrane was assessed by western blot.

**Results:**

Both (-) and (+)-naloxone equally inhibited LPS- and PMA-induced superoxide production with an IC50 of 1.96 and 2.52 μM, respectively. Competitive binding of ^3^H-naloxone with cold (-) and (+)-naloxone in microglia showed equal potency with an IC50 of 2.73 and 1.57 μM, respectively. ^3^H-Naloxone binding was elevated in COS-7 and HEK293 cells transfected with gp91^*phox*^; in contrast, reduced ^3^H-naloxone binding was found in neutrophils deficient in gp91^*phox *^or in the presence of a NOX2 inhibitor. The specificity and an increase in binding capacity of ^3^H-naloxone were further demonstrated by 1) an immunoprecipitation study using gp91^*phox *^antibody, and 2) activation of NOX2 by PMA. Finally, western blot studies showed that naloxone suppressed translocation of the cytosolic subunit p47^*phox *^to the membrane, leading to NOX2 inactivation.

**Conclusions:**

Strong evidence is provided indicating that NOX2 is a non-opioid novel binding site for naloxone, which is critical in mediating its inhibitory effect on microglia overactivation and superoxide production.

## Background

Recent studies strongly support that neuroinflammation plays a critical role in the pathogenesis of various neurodegenerative diseases, including Alzheimer's disease (AD), Parkinson's disease (PD), amyotrophic lateral sclerosis (ALS), multiple sclerosis, Huntington's disease and multiple system atrophy [1]. Microglia, the resident immune cells of the brain, are the major players in the initiation of neuroinflammation and subsequent pathogenesis of neurodegeneration. Once activated, microglia produce and release a variety of proinflammatory factors, such as cytokines, chemokines and reactive free radicals, which contribute to the neurodegenerative process [2]. Thus, anti-inflammatory therapy has been considered a new strategy for disease-modifying intervention.

In the course of developing new anti-inflammatory drugs for PD, we discovered that naloxone, a commonly used antagonist of opioid receptors, was highly effective in preventing dopaminergic neurodegeneration in different rodent PD models by inhibiting inflammatory responses [3-6]. The inhibitory effects of naloxone on inflammatory responses have been reported in our previous studies *in vivo *and *in vitro*. Systemic infusion of 1 mg/kg (+)- or (-)-naloxone significantly reduced intranigral LPS-induced microglial activation and neurotoxicity [5]. In midbrain and cortical neuron-glia cultures, microglial activation and related proinflammatory cytokine production such as nitrite oxide, TNFα and IL-1β, in response to LPS stimulation, were also attenuated by naloxone at 0.1-1 micromolar concentrations [3,4]. Further dose-response study showed a bimodal curve (effective in both micromolar and subpicomolar, but less effective in nanomolar concentrations) for both anti-inflammatory and neuroprotective effects of naloxone [7]. Despite a huge concentration difference, mechanistic studies have revealed that inhibition of microglial superoxide production is the key event underlying naloxone-afforded neuroprotection for both micromolar and subpicomolar concentrations [3,6,7]. Furthermore, we have reported that the neuroprotective effect of naloxone is independent of opioid receptors, since (+)-naloxone, which is an inactive isomer in the activation of opioid receptors, exerts the same potency as the (-)-naloxone in neuroprotection [3-6]. Recent reports from several laboratories have also shown opioid receptor-independent actions of naloxone. Watkins' group reported that (+)-naltrexone, (+)-naloxone and (-)-naloxone, which they showed to be Toll-like receptor 4 (TLR4) antagonists *in vitro *on both stably transfected HEK293-TLR4 and microglial cell lines, suppress neuropathic pain with complete reversal upon chronic infusion [8,9]. In a drug addiction study, Wang *et al. *demonstrated that the acute Gs coupling induced by morphine is completely prevented by co-treatment with both (-) and (+)-naloxone [10]. Similarly, it has been reported that naloxone displays neuroprotective effects in a two-hit seizure model by reducing both cytokine production and microglial activation [11]. Both (-) and (+)-naloxone have also been found to be capable of reducing the severity of aortic atherosclerosis in apolipoprotein-E (apo-E)-deficient mice through inhibition of macrophage activation and superoxide release [12]. Taken together, the non-opioid actions of naloxone may have wide implications in therapy for a variety of diseases. Thus, it is critical to elucidate the novel non-opioid binding site(s) and action mechanisms of naloxone.

The major purpose of this study was to search for the potential site of action mediating the anti-inflammatory and neuroprotective effects of naloxone using a ligand (^3^H-naloxone) binding assay. We have initially attempted to determine both the low (micromloar) and high (subpicomolar) affinity sites of naloxone. However, the specific activity of ^3^H-naloxone is too low to study the binding at subpicomolar concentrations. Thus, the main effort of this study focused on the micromolar concentrations of naloxone. Here, for the first time, we report that gp91^*phox*^, the catalytic subunit of microglial NOX2, is a novel non-opioid binding site of naloxone. Binding of naloxone reduced the translocation of cytosolic subunits to the plasma membrane, leading to inhibition of NOX2 and related superoxide production, which is critical in mediating the anti-inflammatory and neuroprotective effects of naloxone.

## Methods

### Reagents

[^3^H]-naloxone was purchased from Perkin Elmer Life Sciences (Boston, MA, USA). Cell culture reagents were obtained from Invitrogen Life Technologies (Grand Island, NY, USA). Rabbit anti-p47^*phox *^was purchased from Upstate (Billerica, MA, USA). FITC-conjugated goat anti-rabbit immunoglobulin G (IgG) was obtained from Jackson ImmunoResearch Laboratories (West Grove, PA, USA). Rabbit anti-β-actin was obtained from Sigma (St. Louis, MO, USA). Mouse anti-gp91^*phox *^was purchased from BD Transduction Laboratories (San Jose, CA, USA).

### Animals

NOX2-deficient (gp91^*phox-/-*^) and wild type (WT) C57BL/6 J (gp91^*phox*+/+^) mice were obtained from the Jackson Laboratory. All animals were housed in a pathogen free facility with a 12-hour light/12-hour dark cycle and *ad libitum *access to food and water. Housing, breeding and experimental use of the animals were performed in strict accordance with the National Institutes of Health guidelines.

### Primary mesencephalic neuron/glia culture

Briefly, dissociated cells were seeded at 1 × 10^5^/well in poly-D-lysine coated 96-well plates. The cultures were maintained at 37°C in a humidified atmosphere of 5% CO_2 _and 95% air in minimum essential medium (MEM) containing 10% heat-inactivated fetal bovine serum (FBS), 10% heat-inactivated horse serum, 1 g/L glucose, 2 mM L-glutamine, 1 mM sodium pyruvate, 100 μM nonessential amino acids, 50 U/ml penicillin, and 50 μg/ml streptomycin. Seven days later, cultures were used for drug treatments. At the time of treatment, immunocytochemical analysis indicated that the neuron-glia culture consisted of 10% microglia, 50% astrocytes, 40% neurons, and 1% tyrosine hydroxylase (TH)-immunoreactive neurons. For treatment, cultures were changed to treatment medium composed of MEM, 2% FBS, 2% HHS, 2 mM L-glutamine, 1 mM sodium pyruvate, 50 U/ml penicillin, and 50 μg/ml streptomycin.

### Superoxide assay

The production of superoxide was assessed by measuring the superoxide dismutase (SOD) -inhibitable reduction of the tetrazolium salt WST-1. Primary neuron/glia cultures in 96-well plates were washed twice with Hanks' balanced salt solution (HBSS) without phenol red. Cells were then incubated at 37°C for 30 minutes with vehicle control or (-) and (+)-naloxone in HBSS (50 μl/well). Subsequently, 50 μl of HBSS with and without SOD (50 U/ml) was added to each well along with 50 μl of WST-1 (1 mM) in HBSS and 50 μl of vehicle or LPS. The absorbance at 450 nm was read with a SpectraMax Plus microplate spectrophotometer (Molecular Devices) every 2 minutes for 30 minutes. The difference between the absorbances in the presence and absence of SOD was considered to be the amount of produced superoxide.

Preparation, activation, and fractionation of rat blood neutrophils and cell-free assay of NADPH-dependent superoxide production by PMA-stimulated neutrophils membranes.

Neutrophils were purified from fresh blood collected from the abdominal aorta of adult Fischer 344 male rats as described before [13]. Briefly, heparinized blood was sedimented with dextran, and the neutrophils were isolated by centrifugation through the Ficoll-Hypaque density gradient, followed by a hypotonic lysis to remove residual erythrocytes. Purified neutrophils (> 95% viable cells, trypan blue exclusion) were resuspended in warmed phenol red-free HBSS (37°C) and stimulated for 15 minutes with 100 nM PMA or vehicle with gentle agitation. The reaction was stopped by the addition of ice-cold HBSS buffer. Pelleted cells were disrupted and fractionated at 4°C by discontinuous sucrose density gradient sedimentation as described before. Superoxide generation in membrane fractions was measured by monitoring the reduction of ferricytochrome c in a spectrophotometer with the water-jacketed cuvette compartment maintained at 37°C. The reaction was initiated by the addition of 0.2 mM (final concentration) freshly prepared NADPH (tetrasodium salt, Type I, Sigma) into 1.0-ml micro-cuvettes containing detection buffer (100 μM ferricytochrome c, 10 μM FAD, 2 mM MgCl_2_, 2 m NaNs, and 10 mM Hepes, pH 7.4) and 10 μg of membrane. (-) or (+)-Naloxone (a final concentration of 10 μM) or vehicle was added quickly to micro-cuvettes to determine the inhibition of superoxide production. The reference cuvette was supplemented with 600 U/ml SOD (Type I, 3000 U/mg protein, Sigma). Initial rates of superoxide production were calculated from the linear segment of the increase in absorbance at 550 nm and normalized to vehicle-treated control.

### Xanthine/xanthine oxidase reaction

To determine whether naloxone acts as a superoxide scavenger, the superoxide-generating xanthine/xanthine oxidase system was used. Briefly, assays were performed in the presence of indicated concentrations of (-) and (+)-naloxone isomers, 0.01 U xanthine oxidase, 50 μM xanthine, 250 μM partially acetylated WST-1 in 50 mM potassium phosphate buffer (pH 7.6) in a 96-well plate (100 μL/well final volume). Xanthine was added to initiate the reaction, and absorbance at 450 nm was continuously monitored for 5 minutes using a Synergy HT multi-detection microplate reader (Bio-Tek Instruments, Winooski, VT, USA). Results are expressed as a percentage of the increase in absorbance per minute observed with xanthine oxidase only.

### Plasmid transfection

HEK293 cells were maintained in DMEM supplemented with 10% fetal bovine serum at 37°C in a humidified atmosphere of 5% CO_2 _and 95% air. One day before transfection, 1 × 10^6 ^HEK293 cells were seeded into 6-well plates. Flag-gp91^*phox *^and Myc/His-p22^*phox *^plasmids were cotransfected into HEK293 cells using Lipofectamine 2000 (Invitrogen Life Technologies, Grand Island, NY) according to the manufacture's protocol. After 48 hours of expression, cells were collected for binding experiments.

### Isolation of neutrophils from peritoneal cavity

Inflammation was induced by injecting 1 ml of casein solution into the peritoneal cavity of gp91^*phox-/- *^and gp91^*phox+/+ *^mice for overnight and repeat injection the next morning. Three hours after the second injection, mice were euthanized and 5 ml harvest solution (0.02% EDTA in PBS) was injected into the peritoneal cavity. All peritoneal fluids were transferred into 50 ml centrifuge tubes, and then centrifuged at 200 × *g *for 10 minutes. The pellets were washed three times using PBS, and then collected for use [14].

### [^3^H]-naloxone binding assay

Binding experiments were performed according to the methods of Herren, Liu and Zhou [4,15,16]. Briefly, whole cell (2 × 10^6 ^or 4 × 10^6^) or membrane (250 μg) from neutrophils, COS-7, HEK293 or BV2 microglia were incubated with [^3^H]-naloxone (6 nM) plus 10 μM unlabeled naloxone isomers in binding buffer (HBSS + 10% serum) for 2 hours at 4°C. Afterward, the buffer was removed and cells or membrane were washed three times with ice-cold HBSS using 1.0 μm pore size glass fiber filter (Whatman, Florham Park, NJ, USA). Then, cells or membranes were mixed with 10 ml of Ultima Gold scintillation fluid (PerkinElmer, Waltham, MA, USA) and counted for radioactivity. Experiments were performed in triplicate and results are expressed as percentage of total binding observed with corresponding WT group.

### [^3^H]-naloxone immunoprecipitation binding

The ^3^H-naloxone immunoprecipitation binding study was performed by immunoprecipitation using anti-gp91^*phox *^antibody (IP-binding). Specifically, we prepared plasma membranes from wild type COS-7 and COS-7-gp91^*phox*^-p22^*phox *^(COS-7 cells stably expressed with gp91^*phox *^and p22^*phox*^). Membrane pellets were suspended in 0.5 ml IP buffer (10 mM HEPES (pH 7.4), 1% Triton X-100, 5 mM Mg_2_Cl, 10 mM KCl, 1 mM phenylmethylsulfonyl fluoride (PMSF), 1 mM dithiothreitol (DTT), 10% glycerin and protease inhibitor cocktails). After pre-incubation with ^3^H-labeled naloxone (6 nM) in the presence of 10 μM unlabeled naloxone for 30 minutes, membrane aliquots (0.25 mg protein) were incubated overnight with the addition of control immunoglobulin G (IgG) or anti-gp91^*phox *^polyclonal antibody (Santa Cruz Biotech, Santa Cruz, CA) at 4°C. Immunoprecipitation was performed by protein A/G beads (Santa Cruz Biotech). After 4 hours incubation at 4°C, protein beads were washed three times by IP buffer. Then the beads were eluted twice by 0.5 ml glycine buffer (0.1 M at pH 2.5), and radioactivity was counted with a liquid scintillation counter. The binding capacity was determined by subtracting the binding of control IgG from that of gp91^*phox *^antibody.

### Membrane fractionation and western blot analysis

Membrane fractionations were extracted as described previously [17]. BV2 microglia were lysed in hypotonic lysis buffer (1 mM Tris, 1 mM KCl, 1 mM ethylene glycol tetraacetic acid (EGTA), 1 mM ethylenediaminetetraacetic acid (EDTA), 0.1 mM DTT, 1 mM PMSF, and 10 μg/ml) cocktail protease inhibitor incubated on ice for 30 minutes and then subjected to Dounce homogenization (20-25 St, tight pestle A). The lysates were centrifuged at 1,600 × *g *for 15 minutes and the supernatant was centrifuged at 100,000 × *g *for 30 minutes. The pellets solubilized in 1% Nonidet P-40 hypotonic lysis buffer were used as membranous fraction. Equal amounts of protein were separated by 4% to 12% Bis-Tris Nu-PAGE gel and transferred to polyvinylidene difluoride membranes (Bio-Rad, Hercules, CA, USA). Membranes were blocked with 5% nonfat milk and incubated with rabbit anti-p47^*phox *^and mouse anti-gp91^*phox *^(1/1,000 dilution) and HRP-linked anti-rabbit or mouse IgG (1/3000 dilution) for 2 hours. Enhanced chemiluminescence (ECL) reagents (Amersham Biosciences, Piscataway, NJ, USA) were used as a detection system.

### Statistical analysis

The data were presented as the mean ± SE. For multiple comparisons of groups, analysis of variance (ANOVA) was used. Statistical significance between groups was assessed by paired or unpaired Student's *t *test, with Bonferroni's correction. A value of *P *< 0.05 was considered.

## Results

### Naloxone inhibits superoxide production through inhibition of NOX2

**Figure 1:**
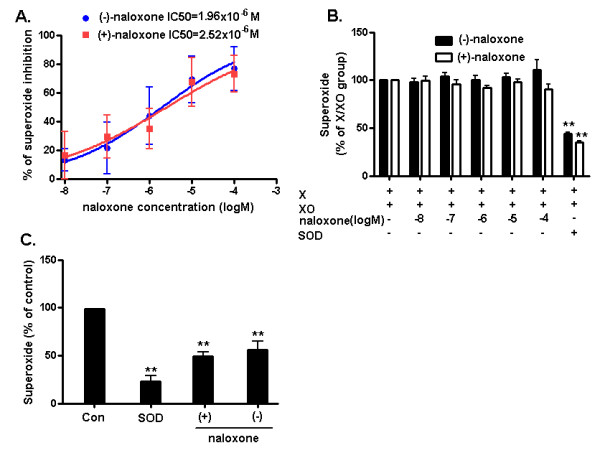
**Naloxone inhibits superoxide production through inhibition of NOX2**. **A**. (-) and (+)-Naloxone isomers are equipotent in inhibiting LPS-induced superoxide production_. _Neuron/glia cultures were pretreated with (-) or (+)-naloxone for 30 minutes before the addition of LPS (15 ng/ml), the production of superoxide was immediately assessed after LPS challenge by measuring SOD-inhibitable reduction of the WST-1. **B**. Both (-) and (+)-naloxone lack superoxide scavenging capacity. Briefly (-) or (+)-naloxone, xanthine oxidase (10 mU), and WST-1 (250 μM) were mixed in potassium phosphate buffer (PBS, 50 mM, pH 7.6). Xanthine (50 μM, final concentration) was added to initiate the reaction (final volume, 1 ml). Absorbance at 450 nm was continuously monitored. **C**. (-) and (+)-Naloxone (10 μM) inhibited NADPH-dependent superoxide production by PMA-stimulated neutrophil membranes in a cell-free system, as measured by the reduction of ferricytochrome c. Results are expressed as a percentage of the control and are mean ± SEM of three independent experiments performed in duplicate. SOD was used as a control to demonstrate its effectiveness to remove superoxide from the system. **P < 0.01 compared with corresponding control group. NOX2, NADPH oxidase; SOD, superoxide dismutase; WST-1, water soluble tetrazolium salt 1; PMA, phorbol myristate acetate.

Although naloxone has been reported to inhibit superoxide production, the detailed mechanism is not known. In the present study, we first demonstrated that superoxide production induced by LPS was significantly inhibited by (-) and (+)-naloxone isomers in a dose-dependent manner with a half maximal inhibitory concentration (IC50) of 1.96 and 2.52 μM, respectively (Figure 1A). These values are much higher than the IC50 for the binding of opioid receptors (in nanomolar), but they are in similar ranges to those previously reported for inhibiting superoxide production in neutrophils [18]. These results also support the contention that naloxone inhibition of superoxide is independent of opioid receptors. Our previous reports indicate that naloxone in these micromolar concentrations was not toxic to cell cultures and no obvious toxic signs were observed in animal studies with a 10 mg/kg/day dosage [3-6]. To rule out the possibility that the reduction of LPS-induced superoxide release was attributed to the superoxide scavenging capacity of naloxone, we evaluated the effect of various concentrations of (-) and (+)-naloxone on superoxide production using the xanthine/xanthine oxidase superoxide-generating system. The increased level of superoxide by the xanthine/xanthine oxidase system was largely inhibited in the presence of SOD (Figure 1), but not by both (-) and (+)-naloxone (0.01 to 100 μM) (Figure 1B), indicating that the naloxone inhibited LPS-induced increase of superoxide from microglia was not due to the superoxide scavenging capacity. Since NOX2 is a major superoxide-generating enzyme in activated immune cells including microglia [19], we then examined whether naloxone directly targeted NOX2 and suppressed superoxide production in a cell-free system. Since it is difficult to generate a sufficient amount of cell membrane from primary microglia for this assay, we used blood neutrophils, which contain a high abundance of NOX2. Neutrophils were first treated with PMA, a widely used agent for superoxide production by stimulating NOX2; and then cell membranes were isolated and used for the measurement of superoxide production by adding NADPH, ferricytochrome c, and FAD. As shown in Figure 1C, both (-) and (+)-naloxone (10 μM) inhibited NADPH-dependent superoxide generation by PMA-stimulated neutrophil membranes. This result indicates a direct inhibitory effect of naloxone on NOX2.

### ^3^H-Naloxone binding is increased in the presence of the gp91^*phox *^membrane subunit

**Figure 2:**
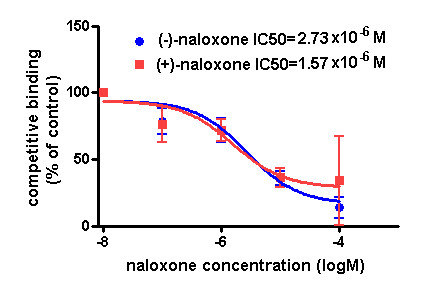
**Displacement of ^3^H-naloxone by both (-) and (+)-naloxone in BV2 microglia**. BV2 microglia (2 × 10^6^) were used for binding experiments. Cells were incubated with hot and indicated fold concentrations of cold naloxone in binding buffer (HBSS + 10% serum) for 2 hours at 4°C. Afterward, the medium was removed and cells or membrane were washed seven times with ice-cold HBSS. Cells were lysed with 400 μl of 1 N NaOH. Cell lysate or membrane was mixed with 10 ml of Ultima Gold scintillation fluid and counted for radioactivity. Results are expressed as percentage of total binding observed with hot naloxone alone and are mean ± SEM of three independent experiments performed in duplicate. **P < 0.01 compared with the corresponding control group, ^##^*P *< 0.01 compared with SOD group. HBSS, hank's buffered salt solution.

The gp91^*phox *^is the catalytic subunit of NOX2 enzyme and mainly locates in the plasma membrane of neutrophils, microglia and macrophages. We hypothesized that naloxone might inhibit the LPS-induced increase in superoxide production by directly binding to the gp91^*phox*^. To test this possibility, ligand binding assays were performed using various cell types containing or lacking the gp91*^phox^*. Initially, BV2 cells, a murine microglia cell line, were used for competition studies. Binding of ^3^H labeled (-)-naloxone was displaced with a wide range of both cold (-) and (+)-naloxone (0.01 μM to 100 μM). As shown in Figure 2, both (-) and (+)-naloxone inhibited ^3^H-naloxone binding to BV2 microglia in a dose-dependent manner with an IC50 of 2.73 and 1.57 μM, respectively. These IC50 values for binding affinity are similar to the IC50 values for inhibiting LPS-induced superoxide production (Figure 1A: 1.92 and 2.56 μM), indicating a well-correlated event between the binding and the inhibition of NOX2. Since the binding affinity of (-)-naloxone toward opioid receptors is approximately 10,000 times higher than that of (+)-naloxone, the equi-potency of these two naloxone isomers in binding to BV2 microglia represents a novel opioid receptor-independent binding site.

**Figure 3:**
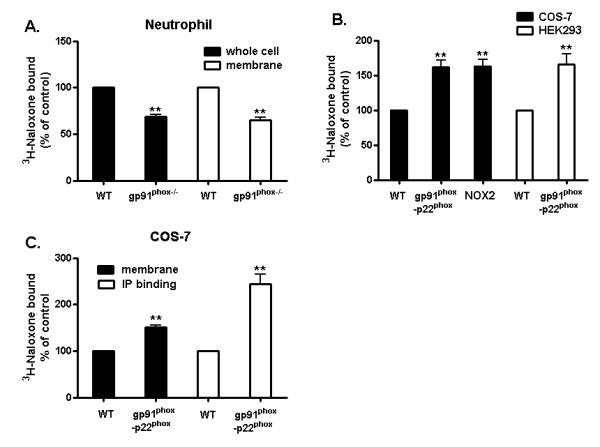
**3H-naloxone binding is increased in the presence of the gp91*^phox^***. **A**. Neutrophils (WT and gp91*^phox-/-^*) whole cell and cell membrane, **B**. COS-7 (WT: transfected with empty vector; gp91*^phox^*-p22*^phox^*: transfected with gp91*^phox^*-p22*^phox ^*subunit and NOX2: transfected with gp91*^phox^*-p22*^phox ^*and cytosolic subunits) and HEK293 (WT and gp91*^phox^*-p22*^phox ^*transfected with gp91*^phox^*-p22*^phox^*, detailed transfection results are shown in Figure 4) were incubated with ^3^H-naloxone (6 nM) in binding buffer (HBSS + 10% serum) for 2 hours at 4°C. Afterward, the buffer was removed and cells or membrane were washed seven times with ice-cold HBSS. **C**. Plasma membranes isolated from COS7 and COS7-gp91*^phox^*-p22*^phox ^*were used to perform the radiolabeled binding directly or to conduct IP using anti-gp91*^phox ^*antibody and the radio-labeled binding assay (IP-binding assay). Then, cell or membrane was mixed with 10 ml of Ultima Gold scintillation fluid and counted for radioactivity. Results are expressed as a percentage of the control and are mean ± SEM of three independent experiments performed in duplicate. **P < 0.01 compared with corresponding WT group. IP, immunoprecipitation; HBSS, hank's buffered salt solution.

**Figure 4:**
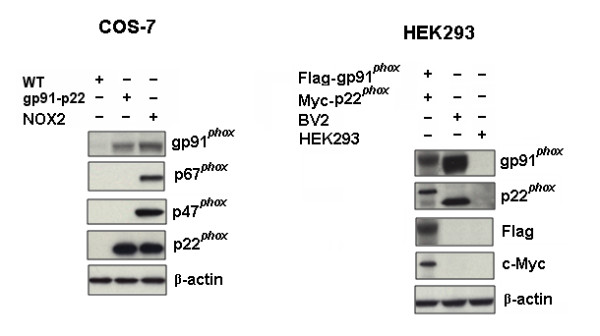
**Gp91*^phox^*-p22*^phox ^*complex is expressed in COS-7 and HEK293 cells**. **A**. Western-blot analysis for gp91*^phox^*, p22*^phox^*, p47*^phox ^*and p67*^phox ^*in COS-7-gp91*^phox^*-p22*^phox ^*and COS-7-NOX2 cells. **B**. Western-blot analysis for gp91*^phox ^*and p22*^phox ^*in HEK293 cells. Briefly, 1 × 10^6 ^HEK293 cells were seeded into 6-well plates. Flag-gp91*^phox ^*and Myc/His-p22*^phox ^*expression plasmids were co-transfected into HEK293 cells using Lipofectamine 2000 according the commercial protocol. After 48 hours of expression, cells were collected for western-blot detection.

To investigate whether naloxone could bind to the gp91*^phox ^*subunit, we performed binding experiments by using neutrophils, which contain the highest level of gp91*^phox ^*among phagocytes, prepared from gp91^*phox*-/- ^and gp91^*phox*+/+ ^(WT) mice. Whole cells or cell membrane fractions (cell free system) prepared from neutrophils were incubated with ^3^H-naloxone for 2 hour at 4°C. The binding capacity of naloxone to the gp91*^phox ^*in WT neutrophils was determined by comparing it with that of gp91*^phox-/- ^*neutrophils. Deficiency in gp91*^phox ^*significantly decreased naloxone binding to neutrophils in both whole cell and cell membranes (Figure 3A). Similar experiments were performed using COS-7 cells stably expressed with the gp91*^phox ^*and p22*^phox ^*(COS-7-gp91*^phox^*-p22*^phox^*) or in combination with p47*^phox ^*and p67*^phox ^*(COS-NOX2) (Detailed transfection results are shown in Figure 4). The reason for the co-transfection of gp91*^phox ^*and p22*^phox ^*is that p22*^phox ^*facilitates stable expression of gp91*^phox ^*in the plasma membrane of COS7 cells [20]. As shown in Figure 3B, ^3^H-naloxone binding capacity was much higher in gp91*^phox^*-transfected cells compared with empty vector-transfected COS-7 cells, which do not express gp91*^phox^*. Transient expression of gp91*^phox ^*in HEK293 cells was also found to elevate naloxone binding (Figure 3B). It should be noted that the decrease of naloxone binding in gp91*^phox ^*deficiency neutrophils was about 40% of total binding, the remaining 50% to 60% was attributed to nonspecific binding. This result is consistent with a previous report by Simpkins *et al. *showing about 60% nonspecific ^3^H-naloxone binding in human neutrophils [18]. The presence of opioid receptors in phagocytes [21] and the low binding affinity of ^3^H-naloxone to the gp91*^phox ^*subunit may explain the relatively high non-specific binding in ours and Simpkins' report. To insure the specificity of naloxone binding to gp91*^phox^*, we have further performed an immunoprecipitation study using anti-gp91*^phox ^*antibody. A much higher increase (2.5 fold) in the binding of ^3^H-naloxone to immunoprecipitated COS7-gp91*^phox^*-p22*^phox ^*membrane proteins was observed compared with immunoprecipitated COS-7 WT membrane proteins with no gp91*^phox ^*(Figure 3C). Together, these binding results imply that naloxone could directly interact with the gp91*^phox^*-p22*^phox ^*complex, and specially, subunit gp91*^phox^*.

### NOX2 activation by PMA enhances the binding capacity of naloxone

**Figure 5:**
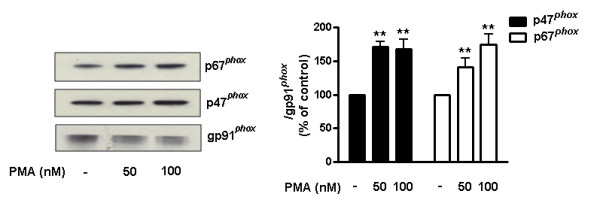
**PMA induces p47*^phox ^*and p67*^phox ^*translocation to the membrane in COS-7-NOX2 cells**. COS-7-NOX2 cells were pretreated with the indicated concentrations of PMA for 20 minutes. Membrane fractions were isolated to perform western blot analysis. Results are expressed as a percentage of the control and are mean ± SEM of three independent experiments performed in duplicate. **P < 0.01 compared with the corresponding control group. PMA, phorbol myristate acetate.

**Figure 6:**
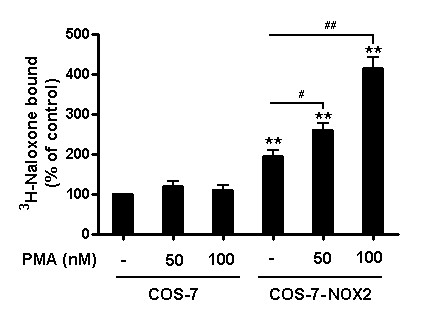
**NOX2 activation by PMA enhances the binding capacity of naloxone**. COS-7-NOX2 (stably expression of NOX2 enzyme) cells were pretreated with the indicated concentrations of PMA for 20 minutes. Then cells were used for binding experiments. After incubation with ^3^H-naloxone for 2 hours at 4°C, cells were mixed with 10 ml of Ultima Gold scintillation fluid and counted for radioactivity. The binding data were normalized by protein concentrations. Experiments were performed in triplicate and results are expressed as percentage of total binding observed with WT COS-7 alone. **P < 0.01 compared with corresponding WT group; ^#^*P *< 0.05 and ^##^*P *< 0.01 compared with and without the PMA treatment group, respectively. PMA, phorbol myristate acetate.

The above-described naloxone binding studies were performed while NOX2 was in the resting stage. Additional experiments were performed to determine the binding capacity of naloxone while the enzyme was in the activated stage. For this purpose, PMA, a widely used compound for the activation of NOX2, was used. It is known that through the activation of PKC, PMA induces the translocation of the cytosolic subunits to the membrane and increases the production of superoxide in phagocytes. In the present study, we examined the binding capacity of naloxone to gp91*^phox ^*after PMA treatment in COS-7-NOX2 cells. PMA treatment activated NOX2 by inducing the membrane translocation of cytosolic subunits (Figure 5). As shown in Figure 6, naloxone binding was elevated in PMA-treated COS-7-NOX2 cells compared to the vehicle treatment. PMA treatment had no influence on background binding of naloxone to COS-7 cells. These results suggest that changes in the conformation resulted from the binding of cytosolic subunits to the gp91*^phox^*, rendering NOX2 in the activated stage, may enhance the binding of naloxone.

### Diphenyliodonium (DPI), a NOX2 inhibitor, reduces ^3^H-naloxone binding

**Figure 7:**
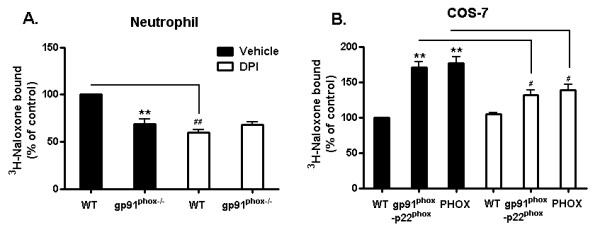
**Diphenyliodonium (DPI), a NOX2 inhibitor, reduces ^3^H-naloxone binding**. **A**. Neutrophil (WT and gp91*^phox-/-^*) and **B**. COS-7 (WT: transfected with empty vector, gp91*^phox^*-p22*^phox^*: transfect with gp91*^phox^*-p22*^phox ^*subunit and NOX2: transfected with gp91*^phox^*-p22*^phox ^*and cytosolic subunits, detailed transfection results are shown in Figure 4) were incubated with ^3^H-naloxone (6 nM) and DPI in binding buffer (HBSS + 10% serum) for 2 hour at 4°C. Afterward, the buffer was removed and cells or membrane were washed seven times with ice-cold HBSS. Then, cells or membranes were mixed with 10 ml of Ultima Gold scintillation fluid and counted for radioactivity. Results are expressed as a percentage of the control and are mean ± SEM of three independent experiments performed in duplicate. **P < 0.01 compared with corresponding control group, ^## ^*P *< 0.01 compared with NO DPI group.

It was reported that DPI binds to the redox core, such as photoreduced flavin or heme of the gp91*^phox^*, and subsequently prevents electron transport [22,23]. Our results showed that DPI pretreatment reduced the binding of naloxone in WT neutrophils and the capacity of naloxone binding became the same between WT controls and the gp91^*phox*-/- ^group (Figure 7A). DPI failed to attenuate ^3^H-naloxone binding to gp91*^phox-/- ^*neutrophils. Similar results were also demonstrated in COS-7 cells stably expressed with the gp91*^phox^*-p22*^phox ^*complex with or without the cytosolic subunits. The increases in ^3^H-naloxone binding in transfected cells were not observed in DPI-treated groups (Figure 7B). These data suggest a possible competition between naloxone and DPI. However, it is important to note that it is not clear from this study whether naloxone and DPI compete for the same binding site. It is possible that naloxone and DPI have different binding sites on gp91*^phox^*. Conformational changes of gp91*^phox ^*after DPI binding may reduce naloxone's binding to gp91*^phox^*. It remains to be determined whether naloxone binds to heme, the C-terminal flavin domain, or any specific domain of gp91*^phox^*.

### Pre-treatment of naloxone inhibits translocation of the NOX2 p47^phox ^cytosolic subunit to membrane

**Figure 8:**
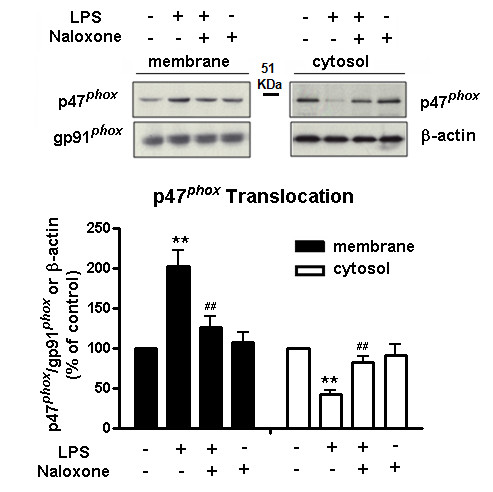
**Pre-treatment with naloxone inhibits translocation of the NOX2 p47*^phox ^*cytosolic subunit to membrane**. BV2 microglia were pretreated with vehicle or naloxone for 30 minutes, followed by LPS treatment for 15 minutes. Membrane and cytosolic fractions were isolated to perform western blot analysis using p47*^phox ^*and gp91*^phox ^*antibodies. Results are expressed as a percentage of the control and are mean ± SEM of three independent experiments performed in duplicate. **P < 0.01 and ^##^*P *< 0.01 compared with corresponding WT and LPS treatment group, respectively; LPS, lipopolysaccharide.

NOX2 consists of the membrane catalytic subunits gp91*^phox ^*and p22*^phox^*, together with several cytosolic regulatory subunits, p47*^phox^*, p40*^phox^*, p67*^phox ^*and a small GTPase RAC [24]. It has previously been shown that activation of the NOX2 enzyme requires that the cytosolic component p47*^phox ^*be phosphorylated and subsequently translocated along with the p67*^phox ^*component to the plasma membrane, where they associate with the gp91*^phox ^*component to assemble into an active enzyme complex. To determine how naloxone inhibited NOX2 activity after binding to the gp91*^phox^*, we investigated the membrane translocation of p47*^phox ^*by determining the cytosolic and membrane levels of p47*^phox^*. BV2 microglia were pretreated with vehicle or naloxone for 30 minutes, followed by LPS treatment for 15 minutes. Western blot analyses showed an increase in p47*^phox ^*levels in the membrane fraction of BV2 microglia 10 minutes after LPS exposure, which was prevented by naloxone pretreatment (Figure 8). At the same time, the decrease of p47*^phox ^*levels in the cytosolic fraction of BV2 microglia induced by LPS was also attenuated by naloxone. Together, these results suggest that binding of naloxone to the gp91*^phox ^*may change the conformation of this membrane component, preventing the membrane translocation of cytosolic subunits of NOX2.

### Post-treatment of naloxone is still capable of inhibiting pre-existing increased binding of p47^phox ^in membrane elicited by pre-treatment of LPS

**Figure 9:**
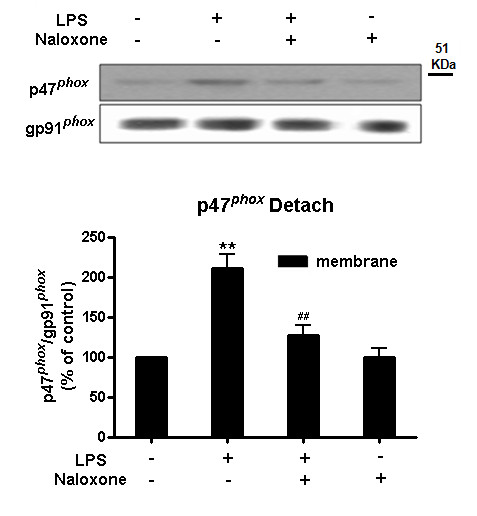
**Post-treatment with naloxone is still capable of inhibiting pre-existing increased binding of p47*^phox ^*in membrane elicited by pre-treatment of LPS**. BV2 microglia were pretreated with vehicle or LPS for 15 minutes. Membrane fractions were isolated and incubated with vehicle or naloxone for 30 minutes at 37°C. Then, the reaction mixture was centrifuged at 100,000 × *g *for 30 minutes. The pellet was isolated to perform western blot analysis using p47*^phox ^*and gp91*^phox ^*antibodies, gp91*^phox ^*as an internal membrane control. Results are expressed as a percentage of the control and are mean ± SEM of three independent experiments performed in duplicate. **P < 0.01 and ^##^*P *< 0.01 compared with corresponding WT and LPS treatment group, respectively. LPS, lipopolysaccharide.

Results from the previous figure (Figure 8) indicate that pre-treatment with naloxone inhibits LPS-induced superoxide and that this is associated with reduction of translocation of cytosolic subunits to the plasma membrane. It is interesting to note that our previous report indicated that post-treatment with naloxone is also partially effective in preventing LPS-induced DA neuron damage [25]. To explain this post-treatment phenomenon, we hypothesize that naloxone is also capable of attenuating NOX2 activity by reducing the amount of membrane-bound p47 and other cytosolic subunits produced by LPS. For this purpose, an experiment using a post-treatment of naloxone paradigm was conducted by preparing membrane fractions from BV2 microglia, which were pre-treated with LPS or vehicle for 15 minutes, and then incubated with or without naloxone. As shown in Figure 9, pre-treatment of LPS activated NOX2 enzyme by inducing translocation to the membrane of the cytosolic subunit p47*^phox^*. After 30 minutes of naloxone post-treatment, the levels of p47*^phox ^*in the membrane significantly decreased, which suggests naloxone counteracted the membrane translocation of the cytosolic subunit p47*^phox^*. These results suggest that naloxone can inhibit the activated NOX2 enzyme by reducing the binding of cytosolic subunits to the membrane.

## Discussion

**Figure 10:**
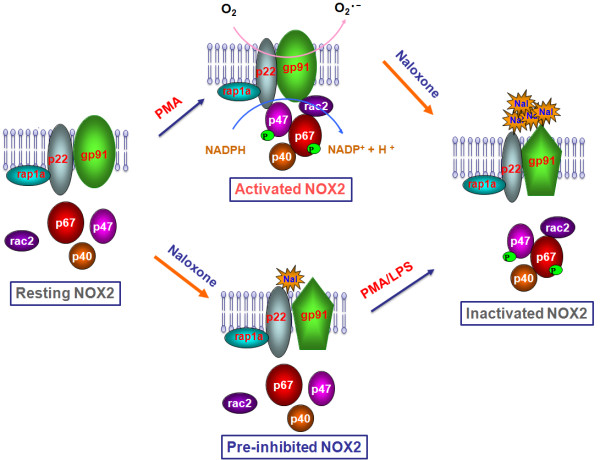
**Summary of the potential mechanism of NOX2 inhibition by naloxone**. Naloxone (Nal) inhibits activation of NOX2 and superoxide production by directly binding to the gp91*^phox^*-p22*^phox ^*complex, and then preventing the translocation of cytosolic subunits. It is interesting to note that the binding capacity of ^3^H-naloxone to the gp91*^phox^*-p22*^phox ^*complex was greatly enhanced after activation of NOX2. Binding of naloxone to the gp91*^phox^*-p22*^phox ^*complex may change the conformation of the protein complex and subsequently decrease the affinity of binding of the cytosolic subunits (p47*^phox^*, p67*^phox^*).

In this study, we demonstrate a non-opioid novel naloxone binding site, which is critical in mediating its anti-inflammatory actions. The salient features of our findings are: 1) both (-) and (+)-naloxone inhibit LPS- and PMA-induced superoxide production in neuron-glia cultures and neutrophil membranes, respectively. In contrast, both isomers failed to reduce superoxide produced by the xanthine/xanthine oxidase system, indicating that the inhibition of superoxide production by naloxone is not due to its superoxide free radical scavenging effect; 2) specific binding of ^3^H-naloxone to gp91*^phox^*, the membrane subunit of NOX2, was demonstrated by an immunoprecipitation study using gp91*^phox ^*antibody, and the binding affinity was further enhanced after NOX2 was activated; 3) Pre-treatment with naloxone prevented the translocation of cytosolic subunits of NOX2 to the plasma membrane. In addition, post-treatment with naloxone of activated NOX2 reduced the binding of cytosolic subunits with plasma membrane. Taken together, this study provides strong evidence demonstrating that naloxone directly binds to gp91*^phox^*. Decreased NOX2-generated superoxide production was critical in mediating the anti-inflammatory and neuroprotective effects of naloxone (Figure 10).

Several reports have documented that naloxone inhibits superoxide production from microglia and neutrophils in an opioid receptor-independent manner [6,18,26]; however, the underlying mechanism remains unclear. Since naloxone contains a phenolic hydroxyl group, we first assessed the possibility that the reduction in superoxide production is due to its free radical scavenging effect. This possibility was ruled out since both (-)- and (+)-naloxone failed to reduce levels of superoxide produced by the xanthine/xanthine oxidase system (Figure 1). This finding led us to propose the possibility that naloxone acts by interfering with upstream events of superoxide generation. Using different types of phagocytes deficient in the gp91*^phox ^*subunit or non-phagocytes transfected with different subunits of NOX2, we provide clear evidence indicating a direct binding of naloxone to the membrane subunit, gp91*^phox ^*(Figure 3). ^3^H-Naloxone binding capacity was decreased in neutrophils deficient in gp91*^phox^*. In contrast, ^3^H-naloxone binding in COS-7 and HEK293 cells transfected with gp91*^phox ^*was elevated compared with vehicle-transfected controls. After enrichment of gp91*^phox ^*protein from COS-7-gp91*^phox^*-p22*^phox ^*cell membranes by immunoprecipitation using anti-gp91*^phox ^*antibody, the radiolabeled naloxone binding showed greater differences in the presence and absence of gp91*^phox ^*(Figure 3C). Overall, our data show a good correlation between the binding affinity of naloxone to the gp91*^phox ^*and its inhibition of LPS-induced superoxide production with similar IC50 values (around 2 μM, Figures 1A &2).

It is important to note that the micromolar IC50 values reported here are much higher than the reported binding affinity of ^3^H-naloxone to conventional opioid receptors (in nanomolar concentrations) [27]. The question is whether these concentrations are clinically relevant for the anti-inflammatory and neuroprotective effects of naloxone. Earlier reports indicated that micromolar concentrations can be reached both in serum and brains after naloxone treatment. Tepperman *et al. *showed that the peak plasma naloxone concentrations in rats were 2.1 and 17 μM after a single subcutaneous injection of naloxone with doses of 1.0 or 10 mg/kg, respectively. Furthermore, it has been reported that brain levels of naloxone are six to seven times higher than that in plasma [28], which might be attributed to involvement of a pH-dependent and saturable influx transport system in the blood-brain barrier transport of naloxone [29]. Our previous report showed that systemic infusion of naloxone (0.4 to 4 mg/kg/day for two weeks with a mini-osmotic pump) dose-dependently attenuated LPS-induced nigral dopaminergic neuron loss in rats [5], and this further supports this contention.

Although we have identified microglial gp91*^phox ^*as a novel binding site in mediating the anti-inflammatory effect of naloxone, the underlying mechanism of how the binding affects subsequent NOX2 activation remains to be studied. Several studies have reported that naloxone could directly alter membrane characteristics. In a hemorrhagic shock rodent model, Curtis and Lefer demonstrated that naloxone increases circulating lysosomal hydrolase activities and suggested that this action results from stabilization of lysosomal membranes [30]. Similarly, inhibition of Na^+ ^and K^+ ^currents in myelinated nerve fibers of sciatic nerve and membrane potential of some cells have also been reported [31,32]. Thus, we speculated that binding of naloxone to gp91*^phox ^*might change the conformation of the protein complex and subsequently decrease the affinity of binding of the cytosolic subunits (p47*^phox^*, p67*^phox^*). One common approach to determine the binding affinity between the cytosolic and membrane subunits of NOX2 is the measurement of translocation of cytosolic subunits to the membrane. Since it is known that the p67*^phox ^*is the subunit that directly binds to the gp91*^phox ^*protein, it would be desirable to measure the translocation of this subunit. However, the lack of suitable antibodies against p67*^phox ^*prevented us from performing this experiment. For this reason, an antibody against p47*^phox^*, which is also bound to the membrane through formation of a complex with p67*^phox^*, was used.

Our speculation is supported by the finding that naloxone pre-treatment reduces the translocation of p47*^phox ^*(Figure 8). It is of interest to note that post-treatment with naloxone was also capable of reducing the LPS-induced increase in the amount of phosphorylatd p47*^phox ^*in the cell membrane fraction (Figure 9). These results suggest that binding of naloxone to activated NOX2 may alter the conformation of gp91*^phox ^*and subsequently decrease binding affinity between cytosolic subunits and the membrane subunit. Although we do not yet have evidence showing actual conformational change of gp91*^phox ^*after naloxone binding, this finding serves to explain why post-treatment of naloxone is still effective in anti-inflammation and neuroprotection. That conformational changes can also alter the binding capacity of ^3^H-naloxone binding is further illustrated by the finding that binding of ^3^H-naloxone in NOX2-transfected COS-7 cells was greatly enhanced after NOX2 was activated by PMA (Figure 6). Taken together, our data strongly suggest that naloxone alters binding affinity between gp91*^phox ^*and the cytosolic subunits.

The non-opioid actions of naloxone have recently received wide attention mainly due to its potential new indications for a variety of diseases. One of the major advances in this research area is the recognition of an anti-nociceptive action of naloxone. Glial activation has been demonstrated to oppose opioid analgesia and enhance opioid tolerance, dependence, reward and respiratory depression by upregulating the production of proinflammatory cytokines (TNFα, IL-1), pain-relevant neurotransmitters from sensory afferent terminals and the number and/or conductivity of AMPA and NMDA glutamate receptors [33-35]. Naloxone, including both (-) and (+) isomers, has been reported to potentiate morphine analgesia equally by blocking morphine-induced glial activation and consequent increases in anti-analgesic proinflammatory cytokines [8,9,36]. Similarly, hyperalgesia induced by morphine-3-glucoronide (M3G), a major morphine metabolite that has little or no affinity for opioid receptors, is also blocked or reversed by (-) and (+)-naloxone [37]. TLR4 was recently recognized to mediate such effects [38-40]. Experiments are underway in our laboratory to determine whether the anti-inflammatory effect of naloxone mentioned above is due to a direct action on the TLR-4 receptor or is indirect through the inhibition of NOX2 inhibition. We performed a radioligand binding assay with microglia prepared from TLR-4-deficient mice. Preliminary results failed to show a decrease in ^3^H-naloxone binding in TLR-4-deficient microglia compared with wild type controls (Wang *et al.*, preliminary observations). Thus, the inhibitory effect shown on the TLR-4 receptor is likely due to an indirect effect through binding to NOX2. In fact, we have previously reported that naloxone inhibits LPS-induced production of proinflammatory factors, such as TNFα and prostaglandins, through inhibition of NOX2-generated superoxide release [41]. Regardless of the site of action, the potential interactions between TLR-4 and NOX2 can be a critical pathway governing innate immunity and warrant further study.

Naloxone belongs to a class of compounds termed as morphinans, with chemical structures similar to morphine. We have previously reported that a large number of morphinan analogs, such as (-) and (+) morphine [42], dextromethorphan [43,44], 3-hydroxymorphinan [45,46] and sinomenine [47] display potent anti-inflammatory and neuroprotective effects similar to that of naloxone. These morphinan analogs have recently received a great deal of attention due to their new therapeutic implications for a variety of diseases, ranging from neuroprotection, anti-nociception, and bipolar depression to drug addiction. Using both *in vivo *and primary neuron-glia cell cultures derived from gp91*^phox-/- ^*mice, we have previously reported that NOX2 is critical in mediating this morphinans-afforded neuroprotection similar to that of naloxone. Further, our preliminary data show that dextromethorphan is able to bind similarly to what we describe for naloxone in this paper, suggesting that gp91*^phox ^*might be a common binding site for the above-mentioned morphinans in their anti-inflammatory property (unpublished data).

## Conclusions

In summary, we provided strong evidence indicating that NOX2 is a non-opioid novel binding site for naloxone, which is critical in mediating its inhibitory effect on microglia overactivation and superoxide production. Although the new implications elicited by the mechanisms of morphinan analogs remain to be studied, the identification of this novel non-opioid binding site mediating the important anti-inflammatory action of naloxone should provide new insights into further understanding the mechanism of action of other morphinan analogs and help better design new drugs targeting this important free radical-producing enzyme.

## Abbreviations

Aβ: β-amyloid; AD: Alzheimer's disease; ALS: amyotrophic lateral sclerosis; apoE: apolipoprotein-E; DPI: diphenyleneiodonium;DTT: dithiothreitol; EDTA: ethylenediaminetetraacetic acid; EGTA: ethylene glycol tetraacetic acid; FBS: fetal bovine serum; HBSS: hank's buffered salt solution; IP: immunoprecipitation; IL-1β: interleukin 1 β; LPS: lipopolysaccharide; MEM: minimum essential media; M3G: morphine-3-glucoronide; NO: nitric oxide; NOX2: NADPH oxidase; PD: Parkinson's disease; PMSF: phenylmethylsulfonyl fluoride; PMA: phorbol myristate acetate; SDS-PAGE: sodium dodecyl sulfate polyacrylamide gel electrophoresis; SOD: superoxide dismutase; TH: tyrosine hydroxylase; TLR4: Toll-like receptor 4; TNFα: tumor necrosis factor α; WST-1: water soluble tetrazolium salt 1.

## Competing interests

The authors declare that they have no competing interests.

## Authors' contributions

JSH and QSW carried out the experimental design. QSW performed the 3H-naloxone binding and western blot experiments and wrote the manuscript. HMG performed the cell free superoxide study. HZ, HMG, SHH, CHC and BW participated in the binding experiment. HZ and HMG helped to edit the manuscript. All authors read and approved the final manuscript.
